# Data concerning the psychometric properties of the “Profile of Emotional Competence” questionnaire administered to a sample of athletes and non-athletes

**DOI:** 10.1016/j.dib.2018.03.067

**Published:** 2018-03-22

**Authors:** Hajer Aouani, Maamer Slimani, Sabeur Hamrouni, Nicola Luigi Bragazzi, Mohamed Elloumi

**Affiliations:** a High Institute of Sport and Physical Education of Ksar Said, Manouba University, Tunisia; b Research Laboratory ‘‘Sport Performance Optimization’’, National Centre of Medicine and Science in Sport (CNMSS), El Menzah, Tunisia; c School of Public Health, Department of Health Sciences (DISSAL), Genoa University, Genoa, Italy; d Department of Neuroscience, Rehabilitation, Ophthalmology, Genetics, Maternal and Child Health (DINOGMI), Section of Psychiatry, Genoa University, Genoa, Italy

**Keywords:** Questionnaire administration, Psychometric properties, “Profile of Emotional Competence” questionnaire, Emotional intelligence in athletes

## Abstract

Emotional Intelligence (EI) can be defined as an “ability to monitor one's own and others' feelings and emotions, to discriminate among them and to use this information to guide one's thinking and actions” (Salovey and Mayer, 1990) [1]. As such EI plays a major role in sport sciences. Studies conducted so far have shown contrasting findings. The paper contains data concerning the psychometric properties of the “Profile of Emotional Competence” questionnaire administered to a sample of 479 subjects (239 athletes, 240 non-athletes, age ranging from 12 to 18 years old, 239 men, and 240 women) in order: i) to explore EI between athletes and non-athletes students and ii) to examine differences in EI of young participants in terms of gender and age.

**Specifications Table**

**Table:** 

Subject area	*Sports sciences*
More specific subject area	*Sports psychology*
Type of data	*Tables*
How data was acquired	*Administration of the Profile of Emotional Competence (PEC) questionnaire to a sample of athletes and non-athletes*
Data format	*Raw and Analyzed*
Experimental factors	*Data were obtained by means of the administration of the PEC questionnaire*
Experimental features	*Descriptive statistics of the PEC questionnaire (mean, standard deviation, kurtosis and skewness), reliability statistics (item-total correlation and Cronbach's alpha coefficient in case of item exclusion), regression analyses*
Data source location	*Tunisia*
Data accessibility	Data are within this article.

**Value of the data**

• These data could be further statistically replicated, and eventually pooled together with other similar data, in order to explore the role of emotional intelligence among athletes, a topic often overlooked in the extant scholarly literature.
• These data could be useful for sports managers, coaches, and athletes, as well as for sports scientists and researchers, for designing and implementing *ad hoc* training programs and interventions, taking into proper account emotional aspects.

## 1 Data

Emotional Intelligence (EI) can be defined as an “ability to monitor one's own and others' feelings and emotions, to discriminate among them and to use this information to guide one's thinking and actions” [1]. As such EI plays a major role in sport sciences. Studies conducted so far have shown contrasting findings. This paper contains data concerning the psychometric properties of the “Profile of Emotional Competence” (PEC) questionnaire, developed by Brasseur et al. in 2013 [2], here administered to a sample of 239 Tunisian athletes *versus* 240 non-athletes, in order to: i) explore EI between athletes and non-athletes students and ii) to examine differences in EI of young participants in terms of gender and age.

General descriptive characteristics of each item and of each sub-scale of the PEC questionnaire are reported in Table 1, Table 2, respectively. Reliability statistics (item-total correlation and Cronbach's alpha coefficient in case of item exclusion) are shown in Table 3. Regression analyses for the sub-scales of the interpersonal and intrapersonal domains are shown in Table 4, Table 5, respectively, whereas regression findings for summary scores (global, interpersonal and intrapersonal domains) are reported in Table 6.

**Table 1:** Descriptive statistics of the “Profile of Emotional Competence” questionnaire.

**Item**	**Mean**	**Standard deviation**	**Skewness**		**Kurtosis**	
			**Statistics**	**Standard error**	**Statistics**	**Standard error**
Item 1	3.157	1.596	−0.202	0.112	−1.514	0.223
Item 2	3.152	1.622	−0.201	0.112	−1.558	0.223
Item 3	2.992	1.692	−0.060	0.112	−1.692	0.223
Item 4	3.856	1.387	−0.984	0.112	−0.372	0.223
Item 5	3.253	1.565	−0.286	0.112	−1.448	0.223
Item 6	4.090	1.378	−1.295	0.112	0.196	0.223
Item 7	3.933	1.384	−1.044	0.112	−0.290	0.223
Item 8	3.365	1.519	−0.371	0.112	−1.332	0.223
Item 9	3.207	1.585	−0.235	0.112	−1.493	0.223
Item10	3.221	1.606	−0.233	0.112	−1.531	0.223
Item 11	3.653	1.345	−0.677	0.112	−0.704	0.223
Item12	3.393	1.594	−0.413	0.112	−1.443	0.223
Item13	3.798	1.341	−0.698	0.112	−0.891	0.223
Item 14	3.547	1.388	−0.586	0.112	−0.948	0.223
Item 15	3.349	1.621	−0.289	0.112	−1.571	0.223
Item 16	3.631	1.448	−0.712	0.112	−0.888	0.223
Item 17	3.420	1.612	−0.436	0.112	−1.433	0.223
Item 18	3.188	1.590	−0.226	0.112	−1.498	0.223
Item 19	3.850	1.387	−1.052	0.112	−0.213	0.223
Item 20	3.238	1.633	−0.269	0.112	−1.549	0.223
Item 21	3.505	1.578	−0.547	0.112	−1.282	0.223
Item 22	3.902	1.374	−1.037	0.112	−0.213	0.223
Item 23	3.814	1.312	−0.776	0.112	−0.674	0.223
Item 24	3.547	1.458	−0.697	0.112	−0.880	0.223
Item 25	2.975	1.620	−0.001	0.112	−1.600	0.223
Item 26	3.198	1.609	−0.228	0.112	−1.525	0.223
Item 27	3.347	1.595	−0.371	0.112	−1.448	0.223
Item 28	3.163	1.610	−0.212	0.112	−1.534	0.223
Item 29	3.079	1.564	−0.129	0.112	−1.484	0.223
Item 30	3.495	1.479	−0.592	0.112	−1.055	0.223
Item 31	3.384	1.568	−0.427	0.112	−1.364	0.223
Item 32	3.668	1.488	−0.792	0.112	−0.833	0.223
Item 33	3.679	1.458	−0.746	0.112	−0.873	0.223
Item 34	3.061	1.551	−0.108	0.112	−1.472	0.223
Item 35	3.572	1.301	−0.531	0.112	−0.785	0.223
Item 36	3.188	1.584	−0.216	0.112	−1.493	0.223
Item 37	3.054	1.578	−0.077	0.112	−1.527	0.223
Item 38	3.336	1.546	−0.369	0.112	−1.367	0.223
Item 39	3.457	1.509	−0.473	0.112	−1.253	0.223
Item 40	2.912	1.559	0.030	0.112	−1.509	0.223
Item 41	3.672	1.449	−0.828	0.112	−0.669	0.223
Item 42	3.374	1.555	−0.397	0.112	−1.355	0.223
Item 43	3.138	1.605	−0.162	0.112	−1.550	0.223
Item 44	3.150	1.557	−0.162	0.112	−1.480	0.223
Item 45	4.006	1.334	−1.179	0.112	0.091	0.223
Item 46	3.000	1.613	−0.048	0.112	−1.586	0.223
Item 47	3.520	1.497	−0.523	0.112	−1.207	0.223
Item 48	3.804	1.298	−0.830	0.112	−0.400	0.223
Item 49	3.253	1.562	−0.283	0.112	−1.437	0.223
Item 50	3.714	1.482	−0.851	0.112	−0.738	0.223

**Table 2:** Descriptive statistics of each sub-scale of the “Profile of the Emotional Competence” questionnaire.

**Sub-scale**	**Mean**	**Standard deviation**	**Skewness**		**Kurtosis**	
			**Statistics**	**Standard error**	**Statistics**	**Standard error**
Identification interpersonal	3.603	0.870	−0.381	0.112	−0.580	0.223
Identification intrapersonal	3.314	1.002	−0.239	0.112	−0.605	0.223
Comprehension interpersonal	3.173	1.013	−0.154	0.112	−0.820	0.223
Comprehension intrapersonal	3.369	0.953	−0.092	0.112	−0.922	0.223
Expression interpersonal	3.294	1.070	−0.220	0.112	−0.818	0.223
Expression intrapersonal	3.474	0.993	−0.390	0.112	−0.637	0.223
Regulation interpersonal	3.393	0.896	−0.218	0.112	−0.692	0.223
Regulation intrapersonal	3.593	0.891	−0.500	0.112	−0.362	0.223
Utilization interpersonal	3.567	0.870	−0.287	0.112	−0.755	0.223
Utilization intrapersonal	3.471	0.901	−0.336	0.112	−0.392	0.223

**Table 3:** Item-total correlation and Cronbach's alpha in case of item exclusion for the “Profile of Emotional Competence” questionnaire.

**Item**	**Item-total correlation**	**Cronbach's alpha in case of item exclusion**
Item 1	0.629	0.927
Item 2	0.660	0.927
Item 3	0.253	0.930
Item 4	0.155	0.931
Item 5	0.694	0.927
Item 6	0.333	0.929
Item 7	0.249	0.930
Item 8	0.346	0.929
Item 9	0.681	0.927
Item10	−0.352	0.935
Item 11	0.159	0.931
Item12	0.341	0.929
Item13	0.353	0.929
Item 14	0.084	0.931
Item 15	0.245	0.930
Item 16	0.339	0.929
Item 17	0.359	0.929
Item 18	0.714	0.926
Item 19	0.312	0.930
Item 20	0.662	0.927
Item 21	0.337	0.929
Item 22	0.176	0.931
Item 23	0.319	0.930
Item 24	0.241	0.930
Item 25	0.696	0.927
Item 26	0.679	0.927
Item 27	0.684	0.927
Item 28	0.649	0.927
Item 29	0.680	0.927
Item 30	0.395	0.929
Item 31	0.667	0.927
Item 32	0.480	0.928
Item 33	0.428	0.929
Item 34	0.675	0.927
Item 35	0.317	0.930
Item 36	0.283	0.930
Item 37	0.676	0.927
Item 38	0.706	0.927
Item 39	0.097	0.931
Item 40	0.633	0.927
Item 41	0.276	0.930
Item 42	0.693	0.927
Item 43	0.652	0.927
Item 44	0.689	0.927
Item 45	0.360	0.929
Item 46	0.599	0.927
Item 47	0.428	0.929
Item 48	0.163	0.931
Item 49	0.673	0.927
Item 50	0.315	0.930

**Table 4:** Regression analyses for each sub-scale of the interpersonal domain of the “Profile of Emotional Competence” questionnaire.

**Source**	**Value**	**Standard error**	***T***	**Pr > |*t*|**	**Lower bound (95%)**	**Upper bound (95%)**	**Value**	**Standard error**	**Lower bound (95%)**	**Upper bound (95%)**
**Identification**										
Intercept	3.038	0.071	42.624	**< 0.0001**	2.898	3.178				
Athletes	0.555	0.071	7.785	**< 0.0001**	0.415	0.695	0.319	0.041	0.239	0.400
Males	0.024	0.071	0.331	0.741	−0.116	0.164	0.014	0.041	−0.067	0.094
12–15 years	0.550	0.071	7.712	**< 0.0001**	0.410	0.690	0.316	0.041	0.236	0.397

**Comprehension**										
Intercept	2.584	0.086	29.997	**< 0.0001**	2.415	2.753				
Athletes	0.644	0.086	7.476	**< 0.0001**	0.475	0.813	0.318	0.043	0.234	0.402
Males	0.171	0.086	1.985	**0.048**	0.002	0.340	0.084	0.043	0.001	0.168
12–15 years	0.363	0.086	4.214	**< 0.0001**	0.194	0.532	0.179	0.043	0.096	0.263

**Expression**										
Intercept	2.523	0.086	29.298	**< 0.0001**	2.354	2.692				
Athletes	0.864	0.086	10.039	**< 0.0001**	0.695	1.034	0.404	0.040	0.325	0.484
Males	0.147	0.086	1.703	0.089	−0.023	0.316	0.069	0.040	−0.011	0.148
12–15 years	0.530	0.086	6.155	**< 0.0001**	0.361	0.699	0.248	0.040	0.169	0.327

**Regulation**										
Intercept	3.123	0.078	39.888	**< 0.0001**	2.969	3.277				
Athletes	0.531	0.078	6.783	**< 0.0001**	0.377	0.685	0.297	0.044	0.211	0.383
Males	0.077	0.078	0.979	0.328	−0.077	0.231	0.043	0.044	−0.043	0.129
12–15 years	−0.066	0.078	−0.844	0.399	−0.220	0.088	−0.037	0.044	−0.123	0.049

**Utilization**										
Intercept	2.995	0.071	42.366	**< 0.0001**	2.856	3.134				
Athletes	0.659	0.071	9.320	**< 0.0001**	0.520	0.798	0.379	0.041	0.299	0.459
Males	0.022	0.071	0.307	0.759	−0.117	0.161	0.013	0.041	−0.067	0.092
12–15 years	0.461	0.071	6.520	**< 0.0001**	0.322	0.600	0.265	0.041	0.185	0.345

**Table 5:** Regression analyses for each sub-scale of the intrapersonal of the “Profile of the Emotional Competence” questionnaire.

**Source**	**Value**	**Standard error**	***t***	**Pr > |*t*|**	**Lower bound (95%)**	**Upper bound (95%)**	**Value**	**Standard error**	**Lower bound (95%)**	**Upper bound (95%)**
**Identification**										
Intercept	2.599	0.079	32.941	**< 0.0001**	2.444	2.754				
Athletes	0.959	0.079	12.159	**< 0.0001**	0.804	1.114	0.479	0.039	0.402	0.557
Males	0.137	0.079	1.730	0.084	−0.019	0.292	0.068	0.039	−0.009	0.146
12–15 years	0.335	0.079	4.241	**< 0.0001**	0.180	0.490	0.167	0.039	0.090	0.245

**Comprehension**										
Intercept	2.923	0.080	36.464	**< 0.0001**	2.766	3.081				
Athletes	0.693	0.080	8.648	**< 0.0001**	0.536	0.851	0.364	0.042	0.282	0.447
Males	−0.089	0.080	−1.110	0.268	−0.247	0.069	−0.047	0.042	−0.130	0.036
12–15 years	0.286	0.080	3.571	**0.000**	0.129	0.444	0.150	0.042	0.068	0.233

**Expression**										
Intercept	2.842	0.080	35.348	**< 0.0001**	2.684	3.000				
Athletes	0.838	0.080	10.422	**< 0.0001**	0.680	0.996	0.422	0.041	0.343	0.502
Males	0.016	0.080	0.201	0.840	−0.142	0.174	0.008	0.041	−0.071	0.088
12–15 years	0.408	0.080	5.078	**< 0.0001**	0.250	0.566	0.206	0.041	0.126	0.285

**Regulation**										
Intercept	3.007	0.073	41.083	**< 0.0001**	2.863	3.151				
Athletes	0.656	0.073	8.959	**< 0.0001**	0.512	0.800	0.368	0.041	0.288	0.449
Males	0.082	0.073	1.122	0.262	−0.062	0.226	0.046	0.041	−0.035	0.127
12–15 years	0.434	0.073	5.923	**< 0.0001**	0.290	0.577	0.244	0.041	0.163	0.324

**Utilization**										
Intercept	3.240	0.079	40.762	**< 0.0001**	3.084	3.396				
Athletes	0.432	0.079	5.436	**< 0.0001**	0.276	0.588	0.240	0.044	0.153	0.327
Males	0.177	0.079	2.222	**0.027**	0.020	0.333	0.098	0.044	0.011	0.185
12–15 years	−0.144	0.079	−1.810	0.071	−0.300	0.012	−0.080	0.044	−0.167	0.007

**Table 6:** Regression analyses for the summary scores (global, interpersonal and intrapersonal domains) of the “Profile of Emotional Competence” questionnaire.

**Source**	**Value**	**Standard error**	**t**	**Pr > |*t*|**	**Lower bound**	**Upper bound**	**Normalized value**	**Standard error**	**Lower bound**	**Upper bound**
**Global**										
Intercept	2.887	0.056	51.431	**< 0.0001**	2.777	2.998				
Athletes	0.683	0.056	12.169	**< 0.0001**	0.573	0.793	0.475	0.039	0.398	0.552
Males	0.076	0.056	1.357	0.175	−0.034	0.187	0.053	0.039	−0.024	0.130
12–15 years	0.316	0.056	5.622	**< 0.0001**	0.205	0.426	0.219	0.039	0.143	0.296

**Interpersonal**										
Intercept	2.853	0.058	48.772	**< 0.0001**	2.738	2.968				
Athletes	0.651	0.058	11.125	**< 0.0001**	0.536	0.766	0.440	0.040	0.362	0.517
Males	0.088	0.058	1.503	0.133	−0.027	0.203	0.059	0.040	−0.018	0.137
12–15 years	0.368	0.058	6.283	**< 0.0001**	0.253	0.482	0.248	0.040	0.171	0.326

**Intrapersonal**										
Intercept	2.922	0.060	48.753	**< 0.0001**	2.804	3.040				
Athletes	0.716	0.060	11.940	**< 0.0001**	0.598	0.833	0.473	0.040	0.395	0.551
Males	0.064	0.060	1.076	0.282	−0.053	0.182	0.043	0.040	−0.035	0.120
12–15 years	0.264	0.060	4.400	**< 0.0001**	0.146	0.382	0.174	0.040	0.096	0.252

## 2 Experimental design, materials and methods

The protocol of the current study received ethical clearance by the Ethical Committee of the National Centre of Medicine and Science in Sports (CNMSS) of Tunisia before questionnaire administration and data collection. Each participant volunteered to take part into this study, after signing an informed consent.

Means, standard deviations, kurtosis and skewness were computed for each item and sub-scale of the PEC questionnaire. Regression analyses were performed to shed light on the predictors of each sub-scale/domain of the PEC questionnaire.

All statistical analyses were performed using the commercial software Statistical Package for Social Science (SPSS, version 24.0, IBM, Chicago, IL, USA). Figures with a *p*-value < 0.05 were considered statistically significant.

These data could be useful for sports managers, coaches, and athletes, in that they could enhance coaching practices and lead to better performances and results. Psychologists can also use the PEC inventory to distinguish between participants’ gender and age and athletes and non-athletes.

## References

[1] Salovey P., Mayer J. (1990). Emotional intelligence. Imagin. Cogn. Pers..

[2] Brasseur S., Grégoire J., Bourdu R., Mikolajczak M. (2013). The Profile of Emotional Competence (PEC): development and validation of a self-reported measure that fits dimensions of emotional competence theory. PLoS One.

